# Application of Wearable Computer and ASR Technology in an Underground Mine to Support Mine Supervision of the Heavy Machinery Chamber

**DOI:** 10.3390/s22197628

**Published:** 2022-10-08

**Authors:** Paweł Stefaniak, Maria Stachowiak, Wioletta Koperska, Artur Skoczylas, Paweł Śliwiński

**Affiliations:** 1 KGHM Cuprum Research and Development Centre Ltd., Gen. W. Sikorskiego 2-8, 53-659 Wroclaw, Poland; 2 KGHM Polska Miedź S.A., M. Skłodowskiej-Curie 48, 59-301 Lubin, Poland

**Keywords:** voice interface, automatic speech recognition, transcription, augmented reality, self-propelled machine, text mining, wearable computer, underground mining

## Abstract

Systems that use automatic speech recognition in industry are becoming more and more popular. They bring benefits especially in cases when the user’s hands are often busy or the environment does not allow the use of a keyboard. However, the accuracy of algorithms is still a big challenge. The article describes the attempt to use ASR in the underground mining industry as an improvement in the records of work in the heavy machinery chamber by a foreman. Particular attention was paid to the factors that in this case will have a negative impact on speech recognition: the influence of the environment, specialized mining vocabulary, and the learning curve. First, the foreman’s workflow and documentation were recognized. This allowed for the selection of functionalities that should be included in the application. A dictionary of specialized mining vocabulary and a source database were developed which, in combination with the string matching algorithms, aim to improve correct speech recognition. Text mining analysis, machine learning methods were used to create functionalities that provide assistance in registering information. Finally, the prototype of the application was tested in the mining environment and the accuracy of the results were presented.

## 1. Introduction

Automatic speech recognition (ASR) is a technology that allows a computer or other device to interpret human speech, for example for transcription purposes or to provide an alternative method of interaction with the user. It combines knowledge and research in the field of computer science, linguistics, and computer engineering. The applications for speech recognition are very extensive. In practice, they can be divided into three groups:

− Short queries—the computer interacts with the user and by understanding speech, generates an useful answer or action;
− Dictation—speech is converted and saved in writing without any interaction with the user;
− Voice recognition—application analyses the voice to identify individuals (e.g., biometric identification).

The first use of automatic speech recognition dates back to the 1960s [1]. In 1952, Bell Labs demonstrated the automatic recognition of digits when spoken over the telephone. The first practical word recognition engines were then created, mainly for words spoken in isolation (i.e., with a pause after each word). From the very beginning, it was considered how to use the solution for industrial applications [2]. The authors presented the use of ASR in the workplace. It indicated that ASR use is especially useful in places where the employee’s hands are often busy and the surroundings do not allow for the use of the keyboard or other similar devices. There are many examples of ASR applications in aviation in the literature. The authors of [3] described the results of pilot programs related to the application of this type of solution in helicopters. The results were encouraging and the voice applications included: control of communication radios, setting up navigation systems, and Automatic Target Transfer System (ATHS) control, which formats and sends air-to-air and air-to-ground messages. This eliminates the need for long keyboard input. In the article [4], the authors examined how to build effective speech recognition systems in aircraft cockpits. The article [5] showed the results for the application of speech recognition for typical communication in air traffic control. The project included the work of air traffic controllers located on the two control towers of the Barajas airport. Currently, together with ASR, virtual reality technologies are being developed to expand the possibilities of applications. The article [6] presented a study on the assessment of the efficiency and comfort of working with AR glasses in an artificially created environment. The task of the respondents was to complete orders. Currently, the most popular applications of ASR are digital assistants, such as Alexa from Amazon, Siri from Apple, Google Assistant, and Cortana from Microsoft. Their main purpose is to assist in performing or fulfilling basic tasks and answering queries via speech. Thanks to access to information from vast databases and various digital sources, these robots help to solve problems in real time, increasing the user experience and productivity.

Such solutions very often shorten the total time to complete the task. For example, tools such as dictation software can allow people to write around 3000–4000 words of content including articles, speeches, books, notes, or emails in 30 min if they are familiar with the subject. While these tools still do not provide 100% accurate results, they often prove to be very useful, especially in time saving. Unfortunately, attempts to use ASR are associated with the problems that are characteristic of this technology. The accuracy of algorithms has been one of the main challenges of speech recognition for many years. The literature contains a lot of research on the factors influencing the effectiveness of solutions and barriers to the implementation of this type of technology. The main factors that can negatively affect the effectiveness of ASR are:

− Environmental impact—ambient noise is a key challenge, especially in cases of high variance in pronunciation and language problems; in practice, this problem is mitigated by building acoustic and language models in ASR applications [2,7,8,9];
− Dictionary size—the more extensive the user’s word resources, the more errors the system makes, which may be important especially in specialist vocabulary [2,9];
− Strings of characters—very often a keyboard is better in this case [2,10];
− Learning curve—entering text by voice can be simple and fast, but for an inexperienced user it can take much longer than using the keyboard [11,12];
− Individual voice characteristics and tone—The tone of voice often changes between users depending on the time of day, perceptual abilities, or different situations. The correctness of pronunciation and the pitch of the voice are also critical. ASR requires a constant volume to function optimally.

This article will present an example of the use of ASR technology in the difficult conditions of an underground mine. The purpose of ASR is to support the foreman in recording in the MES (software that keeps track of and monitors manufactured raw materials and goods) and ERP (software that manages operational activity such as plant scheduling, order processing, or inventory management) systems the course of work in the heavy machinery chamber [13]. In terms of the development of the voice interface, attention was also paid to a number of disturbances of various origins. First of all, it is necessary to mention the noise coming from the local air conditioning. In addition, one can hear knocks from ongoing repair work, machine engines working, or reversing signals. In the background, conversations of employees occur who move around the foreman’s workplace. This is problematic because the main source of problems currently encountered when using speech recognition is the user’s environment. Another important characteristic of the mining environment is specialized vocabulary. This requires the preparation of an appropriate dictionary, thanks to which the algorithms will not become confused when transmitting voice to text. In article [10] the authors described the main problems related to the difference between merely speaking queries and typing them directly. Voice input reduces some of the typing problems, such as correct spelling (no spelling mistakes, typos), but can pose other challenges, such as poor pronunciation of words. Incorrect pronunciation may lead to the algorithm giving a completely different word. However, the names of things/places/people are still easier to transmit by voice (especially when the native language is used).

Finally, when considering the use of ASR, the need to educate personnel must also be considered. Article [11] contained the results of a study conducted on a group of 28 people who had experience with the use of a fairly outdated ASR system. Not only was the effectiveness examined, but also the satisfaction and subjective impressions of these people. The study draw the conclusion that entering text by voice is simple but may be much slower than entering it using the keyboard, depending on the user experience. Despite the fact that nowadays almost everyone has already had contact with ASR, for example when using a telephone, the initial recognition and conversations with mine workers showed a discrepancy in their acquaintance with ASR. This is largely due to the divergence of generations in different positions. Therefore, in order to properly examine the situation, one must also take into account the need to train staff. The study presented in [12] showed that additional instructions and indicators on the screen shown during the first-time use greatly accelerated learning.

Industrial conditions, especially those observed in the mine, contain most of the potential problems described above. Specialized vocabulary, the constant presence of noise, and limitations of AR equipment in terms of resistance and safety (the equipment must be resistant to mechanical damage, high humidity, temperature, dust, have appropriate approvals ensuring its use in the workplace). Attempts to apply speech recognition in industry must take into account all these limitations [14,15,16].

The article presents all the key functional requirements of the voice interface to handling ERP and MES systems, as well as the system architecture and its key procedures. One of the key challenges in building a voice assistant was the development of a specialized dictionary and dedicated text-mining tools.

## 2. Characteristics of the Heavy Equipment Foreman’s Work

The role of the heavy equipment foreman is a professional one, including managing the operation, servicing, and repair of heavy equipment such as self-propelled machines (loaders, haul trucks, drilling machines, bolting machines). The main responsibilities include: allocations of operators and equipment among various mining areas, ensuring the proper maintenance of all machinery, employee training, warehouse control, parts ordering, and keeping shift records of machines and employees. This position is also the official who is responsible for the health and safety of the employees during the work shift. Among all of the duties mentioned, the most time-consuming is recording the course of the work shift in MES (Manufacturing Execution System) for electronic information flow. Theoretically, all of the necessary documentation can be completed within one hour. In practice, the time of registration extends to the entire shift due to the specific features of this position (multitasking, uninterrupted communication, contact with various places of the mine through various communication channels, high mental stress).

**Figure 1:**
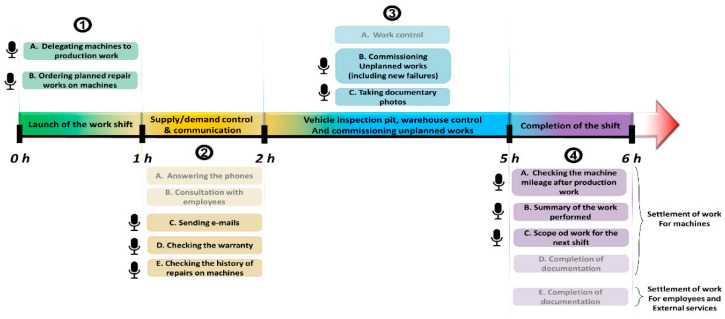
Hourly schedule of the foreman’s work.

The scope of the foreman’s work load depends on mining departments and the number of machines assigned to the chamber and the shift number. The first work shift (the earliest one) is the most overloaded due to commuting and lower costs of external services. A typical work shift lasts about 6 h and can be divided into four main blocks: (1) launch of the work shift; (2) supply/demand control and communication; (3) vehicle inspection pit, warehouse control, and commissioning unplanned works; (4) completion of the shift. Figure 1 shows the course of the foreman’s shift and lists typical tasks in individual time blocks. The microphone icon indicates those tasks that can be improved via the voice interface. It is expected that the implementation of the voice assistant will result in number of benefits such as: time savings; communication improvement; foreman mobility improvement; elimination of typos in the reports as well as errors arising from data entered incorrectly.

At the beginning of the shift, the foreman becomes acquainted with the document of the workload among the machinery and employees on the current shift. After the employees and service technicians are delegated, the foreman goes to his desk, where he conducts further communication and begins to record in the field of delegating machines to production work and ordering planned repair works on machines. The next block of the work shift concerns the current demand and supply control as well as internal and external communication. Now, the current scope of work includes the exchange of telephones (usually several dozen) and consultation with employees. The key is to check the demand for repairs, compare with the machines’ warranty status (machines and components), prioritize maintenance tasks, check the status of parts orders, etc. The implementation of the above tasks also involves writing e-mails and checking the records of orders for parts and materials to the warehouse, maintenance data, and warranty files. In principle, the activities provided in this block may take place until the end of the shift. The next, third block is related to the vehicle inspection pit, warehouse control, and commissioning unplanned works. In this block, the foreman spends a considerable part of his time on revision work on the vehicle inspection pit including acquiring documentation. Very often there are random events related to failures of machines relocated to production, which require an urgent planning of repair work and commissioning the operator to work for the next part of the work shift. In this part of the shift, the foreman also supports the work of employees and performs training. About an hour before the end of the work shift, the fourth block related to completion of the shift begins. The key responsibilities of the foreman are related to the record at the end of the work shift in IT systems and paper documentation, separately the settlement of work for machines and for employees, as well as external services. It is also important to make a list of tasks to be performed on the next shift, which the foreman supervising the next shift is obliged to know and perform.

## 3. MES and ERP Systems Used in Examined Case

As mentioned above, the work process records for a shift in the heavy machinery chamber and the general flow of information are carried out in the KGHM Polish Copper Ltd. mines mainly through MES and ERP systems and, to a lesser extent, in flat files, paper documentation, or an ordinary telephone report. Basically, we can distinguish three main MES/ERP systems:

− CMMS (Computerized Maintenance Management System)—IT support for servicing underground self-propelled machines, including registration of service technicians’ working time and used materials, and recording the scope of the work performed;
− e-Raport—communication and information exchange platform in the following mine areas: mining works department, infrastructure, energy and mechanical department for underground machines;
− SAP HR&MM modules—KGHM’s main transaction system for managing economic processes; the scope of the system available to the foreman includes the HR and payroll (HR) and materials’ management (MM) modules.

In practice, the collection of information in the course of the machine operation and maintenance process has a wide analytical application [17,18]. It allows one to calculate not only mine performance indicators [19], analyze context awareness in predictive maintenance [20], but also machines and operators [21,22]. It can also be used for building complex reliability models for machines and their parts [23,24,25], assessing the service life of selected manufacturers’ parts and estimating downtime or residual life-time of machinery [26,27]. Unfortunately, this requires keeping records in several systems, which significantly reduces the employee’s time for main work duties. For this reason, the main expectations of the mining industry are the implementation of modern IT technologies to support the underground worker and mitigate monotonous and repetitive activities [28,29,30,31].

Each of these systems has its own application which the foreman uses to fill in information from the shift. Information is intertwined, sometimes requiring repetition. The foreman must be present at the computer when refilling. The summary of the tables’ fields that are accessible by the foreman is shown in Table 1, while Table 2 describes in detail the fields that are used by the proposed solution.

## 4. Materials and Methods

In order to build a fully working voice assistant, it was necessary to review the available programming environments and already made ASR tools. From the perspective of the development of prototype application the main emphasis was placed on: (a) open-source solutions that can be freely developed; (b) support for the Polish language; (c) possible integration with Android operating system; (d) low complexity of use; (e) possibility of integration with other text mining tools. The free “SpeechRecognition” library [32] was selected for work due to fulfilling most of the above-mentioned criteria. Speech recognition systems are speaker independent. The model is trained on a large corpus and responds to a word regardless of who speaks. This library implements most of the top solutions (as speech recognition engines), such as: Microsoft Azure Speech; Google Speech Recognition; IBM Speech to Text and many others. For the specific task, the Google engine was used, mostly because of its ability to work well with the Polish language. Unfortunately, even the best speech-to-text algorithms make mistakes, hence the need to use a form of verification.

As measures of effectiveness, the following were proposed: the percentage of exactly translated words, word error rate, the percentage of similarly translated words, Levenshtein Edit Distance (1) [33], Jaro Similarity (2) [34], and the Jaro–Winkler Similarity (3) [35]. The first three of the proposed metrics measure similarity between two texts at the word level while the rest of the metrics works on the phoneme level.

(1) $$lev\left(a,b\right)=\{\begin{array}{c} \;lev\left(tail\left(a\right),\;tail\left(b\right)\right)\;\;\;\;\;\;\;\;\;\;\;\;\;if\;first\;letter\;in\;a\;and\;b\;are\;the\;same\;\\ 1+min\{\begin{array}{c} lev\left(tail\left(a\right),b\right)\\ lev\left(a,\;tail\left(b\right)\right)\\ lev\left(tail\left(a\right),\;tail\left(b\right)\right) \end{array}\;\;\;\;\;\;\;\;\;\;\;\;\;\;\;\;\;\;\;\;\;\;\;\;\;otherwise \end{array}$$

where tail of string a/b is a string of all characters but the first character of a/b.

(2) $$jaro\left(a,b\right)=\{\begin{array}{c} 0\;\;\;\;\;\;\;\;\;\;\;\;\;\;\;\;\;\;\;\;\;\;\;\;\;\;\;\;\;\;\;\;\;\;\;\;\;if\;m=0\\ \frac{1}{3}\left(\frac{m}{\left|a\right|}+\frac{m}{\left|b\right|}+\frac{m-t}{m}\right)\;\;\;\;otherwise\; \end{array}$$

where m is the number of matching characters (two characters from a and b are matching if they are the same and not further than $|\frac{\max\left(\left|a\right|,\left|b\right|\right)}{2}|-1$ characters apart, and t  is the number of transpositions (number of matching characters divided by 2)

(3) jarowinkler(a,b)=jaro(a,b)+lp(1−jaro(a,b))

where jaro(a,b) is the Jaro similarity, l is the length of common prefix at the beginning of the string, up to maximum of four characters and p is the constant scaling factor for how much the score is adjusted upwards for having common prefixes (usually p = 0.1).

The Levenshtein distance counts the number of changes needed to convert the string a to b. The Jaro similarity is a measure of characters in common including transposition. Winkler modified this algorithm to support the idea that the differences near the start of the sequence are more significant than the differences near the end of the sequence. Usually Jaro and Jaro–Winkler similarities are suited better to comparing smaller strings such as words. In this case, we were dealing with the problem of understanding speech rather than diction, so correct results with statistics such as word error rate should be sufficient.

### 4.1. Initial Experiments with ASR

When the ASR method was established, the first tests of the algorithm and the impact of specialized vocabulary were carried out. To compare the results, one needs a separately prepared transcription of the recording text. The algorithm was tested by reading fragments of texts describing the mechanical works performed on machines. In these experiments, different noises were recorded and then added to clean speech. The overall results were satisfactory, yet some mistakes were found. Most of the errors were due to the specialized vocabulary. To deal with this problem, it was proposed to use a specialized dictionary that would match misread words from speech by similarity. With the support of the CMMS database such a dictionary was created.

Another problem is the matching of caught names to their full versions in databases. When talking, we often shorten some names, either because of being in a hurry or because of the difficulty of pronouncing the name. For example, when employees talk about the machine “123C” they really mean machine “AB-123C”. When we catch that it is a machine, as in this example, all that remains is to match the formal name with the colloquial name. The order is very important here, so it is best to combine them after the longest fragment, which comes down to the longest common subsequence problem. The LCS (Longest Common Subsequence) function is defined as follows (4):

(4) $$LCS=\{\begin{array}{c} 0\;\;\;\;\;\;\;\;\;\;\;\;\;\;\;\;\;\;\;\;\;\;\;\;\;\;\;\;\;\;\;\;\;\;\;\;if\;i=0\;or\;j=0\\ LCS\left(A_{i-1},B_{j-1}\right)+1\;\;\;\;\;\;\;\;\;\;\;\;\;\;\;\;\;\;\;\;\;if\;i,j>0\;and\;a_{i}=b_{j}\\ \max\left\{LCS\left(A_{i},B_{j-1}\right),LCS\left(A_{i-1},B_{j}\right)\right\}\;\;\;\;\;\;\;if\;i,j>0\;and\;a_{i}\neq b_{j} \end{array}$$

where A and B are two sequences, and $a_{i},\;\ldots ,\;a_{m}$ or $b_{i},\;\ldots ,\;b_{m}$ are words from those sequences. In this case, it is enough to know which formal name has the highest LCS with a colloquial name to choose it appropriately.

With above described changes, the tests were repeated, and the results are presented in Table 3. For the effectiveness evaluation, the well-caught words factor was used as well as the measures presented earlier in this chapter. The Levenshtein distance was normalized using the length of longer sequence. As one can see, the overall results are good (mostly in range 0.9–1.0).

### 4.2. Processing ASR Results with Text-Mining Techniques

At this point the speech was successfully translated to text, which is unusable for the given task at this moment. First, one needs to relate to the problem of the various forms of every word (which is especially large in Polish language). For this task, two techniques are used in common solutions: stemming and lemmatization. Stemming is truncating words from inflected endings to leave only the root of the word (stem), and lemmatization is changing inflected forms into one basic form (lemma). As an example, consider several variations of the word change: change, changing, changes, changed. In the process of their stemming, the resulting word will be “chang”, but if instead of stemming, the lemmatization were used, the resulting word (lemma) should be their basic form which is “change”. In English, stemming works well in most of the cases, but in Polish, due to the complicated way of creating inflected word forms, lemmatization is a much better choice. In addition, by having a lemmatization tool and access to a database with historical information, one can use it to facilitate the retention of information/notes.

**Figure 2:**
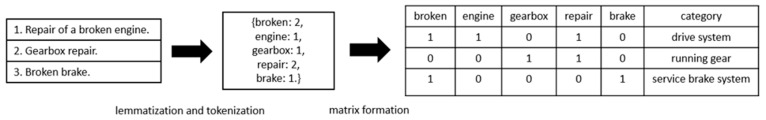
Process of creating a matrix of words.

Notes are difficult to filter and view. Therefore, it would be best to assign some kind of label or categories to them as well. It can be completed manually or automatically using an appropriately built model. Historical entries from the CMMS database with manually assigned categories were used for the model training process. We have tried to assign categories to as many entries as possible with the help of specific expressions. Machine representation of text is at a fundamental level not different from any other variables. When dealing with text data, one needs to represent them with numbers in order to use many of the current day methods. From such entries, a matrix of the most important words was prepared—words selected by their frequency of occurrence. This was achieved done by first performing the tokenization of records, then the selection of keywords and finally form a matrix (with one-hot-encoding) method. The concept is shown at Figure 2.

First, the text describing the machine repair is lemmatized and the stop words are removed. Then, each word for each line is counted and tokenized. This shows the transition between the first and second box. Then, a matrix is created from the collected words. One line describes one commissioned work. Each line has a corresponding category. The key words for a given row are added to columns. Then, the matrix is normalized and the model learns through it how to assign categories. The random forest method was used for classification. In a situation where the model does not find a category of sufficient probability, it assigns the category “other”. The predicted probabilities for the input sample are computed as the average predicted probability of the assigned category by random forest classifier. Random forest is performing well in text classification among other things because it mitigates the inherent challenges involved in textual data such as high dimensionality, sparsity, and noisy feature space [36]. The better the training data, the better the algorithm will assign categories. Historical information describing damages to elements of mining machines was used to train the model. For each entry, a category describing the machine system from which the damaged element comes was matched. The division of categories are presented in Table 4.

Due to the quite large discrepancy in the number of entries for individual categories, attention was paid to keep the class proportions in the training and test set. On the currently used training and testing set, the accuracy of the assigned categories is over 90%. The confusion matrix for such a dataset is presented in Table 5

### 4.3. Voice Assistant

With the support of the foremen involved, and with the use of the above mentioned tools, the voice assistant was developed. During the consultations with workers, it was concluded that the form of voice assistant should be as follows: (1) the assistant waits for one of the task commands to be triggered; (2) there is a short conversation between the worker and the assistant (mainly the worker is giving the assistant the information necessary to complete a given task specified by the command); (3) the assistant goes into stand-by mode, where it analyzes only a small window of time in order to find one of the commands.

**Figure 3:**
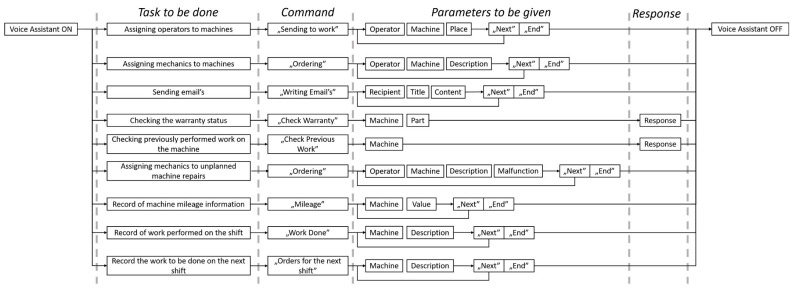
Diagram of commands for main tasks.

In the course of project, the main tasks of the foreman were defined, and then those tasks that could be implemented by the voice assistant were selected. A separate command was created for each of these tasks, and with it a different conversation flow. Those main tasks along with their conversation schema are presented in Figure 3. The overall scheme is similar in most of the tasks:

(1) Worker says the command that defines what he want to do;
(2) Worker says the necessary parameters from parameter–value (e.g., “Operator”, “John Smith”);
(3) Worker is given a choice: either he wants to repeat the same command “next” or exit “end”. Repeating the same command means that the foreman is, for example, sending another operator to work.

**Figure 4:**
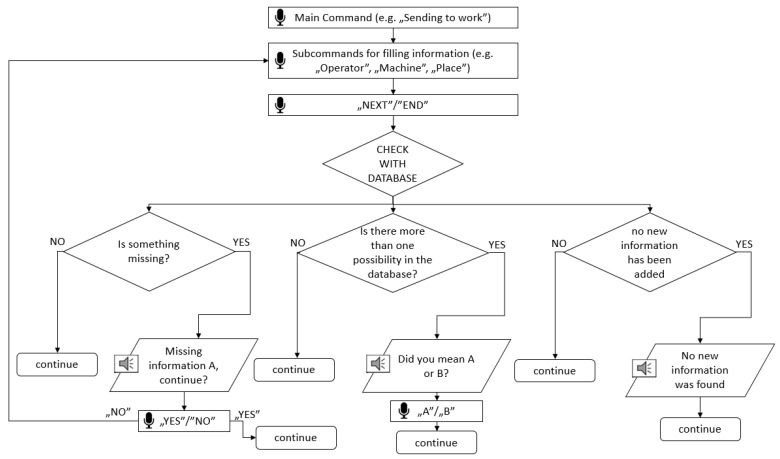
Diagram of the data validation algorithm.

The above mentioned schema (Figure 3) presents the general communication graph. However, with each assistant’s use, there is a risk that some information will not be understood correctly or missing, so to address that, information checking with the database was programmed (Figure 4). Each provision of information is compared with the database and information already completed, and a message is returned depending on the existing problem. Some require a response (such as selecting option, when there is more than one possibility), while others are simply a message to the operator (such as a message that no new information was saved). Finally, all paths end with a “continue” statement which covers all possible next procedures that the assistant performs (filling database records, listening for more information, etc.).

**Figure 5:**
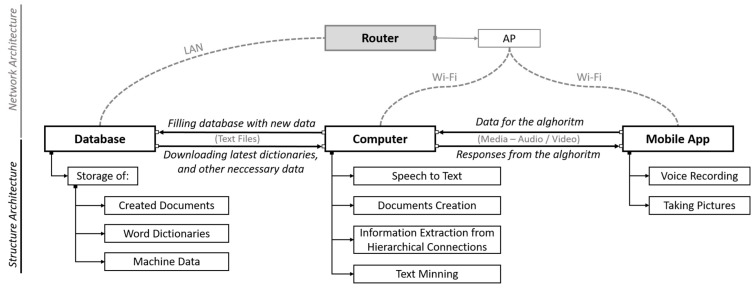
Client-server architecture for the assistant.

Finally, the assistant was implemented in a client-server architecture. The main part of the product (the server) was implemented in Python environment on a computer. To this computer, via wireless means, the clients are connecting. Each of the clients is an Android smartphone device with a special app installed on it (app developed especially for this purpose). The role of the clients is to maintain the communication with its users (record the voice and speak the responses), while the server executes all of the processing. The scheme of this solution is presented in Figure 5.

The early prototype of presented solution was built and implemented in the foreman’s workplace in an underground mine. The mobile application was created in Android in Java. The versions were tested on Android 10.0, but the application was built on the AppCompatActivity class, which allows one to use the newer platform features on older Android devices. For full operation, the application requires access to a microphone, camera, internet, and external storage. They are used for speech transcription, data transmission over the network, and taking photos and recordings. After building the .apk file (format used for package distribution and installation on the Android operating system), the application weighed 3 MB.

## 5. Demonstrator Test in Industrial Conditions

During an organized trip to the mine chamber, tests of the device were carried out. The test included a scenario of sending machines to operation by the foreman on a work shift. For the purposes of the test, sample information was obtained from the already completed work shift. Based on this, a scenario was prepared that included:

− 16 employees;
− 16 machines;
− 5 mining departments.

The provided forms were tested by three people with different experience in operating the developed device (Table 6). The developer of the tool has been working on it for a year and had no problems with using it. A person familiar with the application sometimes had to think about how to properly issue the commands. The operation was explained to a person who had not previously had contact with the application. It took a few minutes and a couple of rehearsals with the recording for them to be able to use the tool. The biggest problem for users was the lack of contact with the names of machines and employees. A special cheat sheet was prepared for this. In practice, mine workers knew this information.

**Figure 6:**
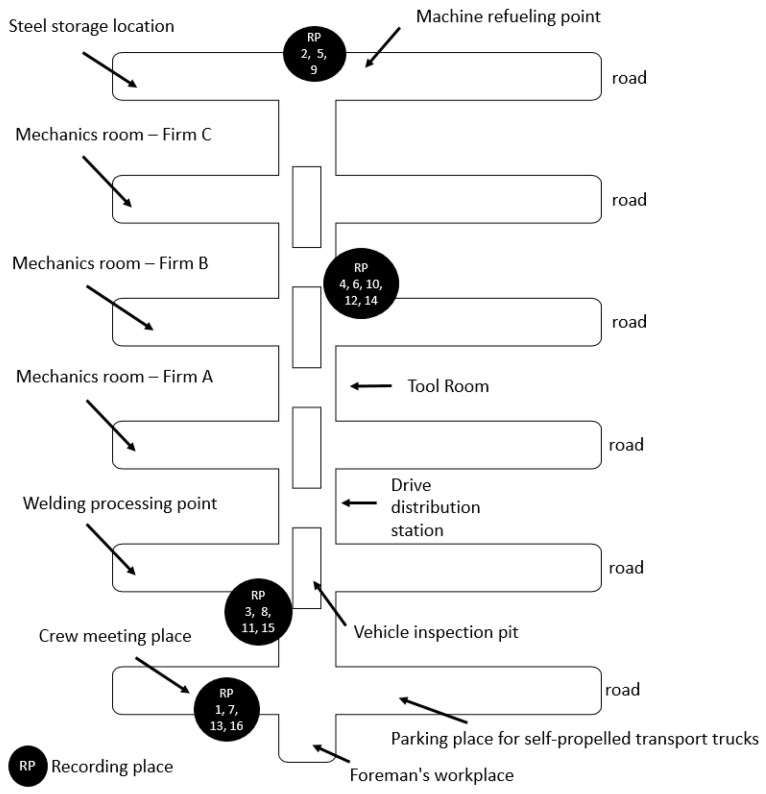
Map of the chamber with marked places of recordings.

Recording was carried out at various locations in the chamber. These places are presented in Figure 6. Different noise levels are possible at each location. The closer to the inspection pits, the greater the chance of loud noises. There are various noises when repairing the machines, from the constant noises of a running machine to sudden high-pitched alarms. It is also worth paying attention to the distance from the router. The presence of a large number of devices and electrical equipment affects the quality of the connection between the client and the server. Devices such as air conditioners or heavy motors can interfere with the signal. These components are mainly placed in machines. Up to two can stand at each inspection pit. The more machines there were between the client and the server, the worse the connection was.

In addition, each of the recorders tried to record at a similar pace and with a different distance from the microphone (close to the face and lying on the table next to the speaker). In the background, you can hear noises resulting from human conversations, ventilation, or working on the machines. Each recording was then run through the application algorithm and then compared with the correct values. The results are presented in Table 7 and Table 8.

On average, about 70–80% of the information for each of the recordings was filled correctly. It can be noticed that the poor results are for the places closer to the machines or mechanics’ workplaces. The worst results are for the recordings where you could hear sudden loud noises in the middle of the recording. Holding the microphone to your face helps in these cases. In situations with constant noise, there are no noticeable differences when having the phone near your face or on the table next to the speaker. Recordings last approximately 2 min on average, which gives 7.5 s to fill one item. This gives similar results to the observed manual filling by the foreman (with an indication that the program was already turned on and opened on the appropriate tab). On average, every second instruction has been fully filled and in the remaining ones it is often only one piece of information missing.

As can be seen in Table 8, the department is usually the missing information. There is quite a difference between the quality of filling the variables such as machine or operator, and the department. This is probably due to the length of the variable—departments are three–four letters long, where other variables are much longer, which can cause a bad fit or a lack of matching to its formal version. In addition, the machine and department contain both letters and numbers. The operator consists only of proper names, which, even if heard inaccurately, are easier to assign to the correct characteristic value.

## 6. Discussion

As can be seen from the tests, it is possible to use speech recognition tools in underground mining conditions. The tests carried out showed about 70–80% correctness when filling in the prepared forms, depending on the place of recording. Sudden high-pitched noises made it difficult to catch some information, but usually, only the short pieces of information. The presence of constant ventilation noise does not interfere with the speech-to-text conversion. Additional work is currently underway to investigate how different noise reduction algorithms affect external noise. Depending on the results from collected data, this can also significantly improve the results for samples with more noise.

Looking at the results from the perspective of improving the foreman’s productivity, positive effects can also be noticed. The recording simulated the standard dispatch of employees on a shift. On average, all took 2 min and for one employee it was about 8 s. It is comparable to the time of filling in information for that one employee manually. However, voice filling skips steps such as starting the appropriate program and loading the appropriate tab (which, depending on the computer, may take up to several minutes). The ability to fill in information anywhere in the chamber reduces the filling time also by not needing to return to the workstation. There remains the issue of the correction of errors and gaps due to inputting information by the ASR. Data gaps are reported at the level of voice assistant and can be completed by the user. Data errors can be checked and corrected when the worker returns to the workstation. With the current accuracy of the application, these are only a few cases that should not extend the data verification performed at the end of the shift. In summary, manual fill-in, which in theory takes a similar amount of time to voice fill-in in practice, takes much longer. The elimination of indirect factors (a need to return to the workstation, delay caused by starting the program) would greatly improve productivity.

## 7. Conclusions

In various fields, including more and more often in industry, solutions using automatic speech recognition are used. The aim of the study was to identify the possibility of using a voice assistant as an aid for a foreman to record work in the x heavy machinery chamber. A thorough examination of the foreman’s workflow showed that he was required to multitask. In addition to numerous regular tasks, there are also random events, the need to continuously support the employees, and control of their safety. All of these are also associated with mental stress. Completing the documentation takes place in the meantime and is interrupted by other tasks. Facilitating this one task would certainly improve the comfort of work, and the time saved could be used to perform other tasks. Recognition of the forms required to be completed by the employee additionally showed that sometimes he has to fill in the same information several times. The use of ASR based on simple commands would facilitate this process in several aspects: the ability to enter information from any place (not only at the computer), no repetition when entering information, the ability to save information immediately after issuing the order (no problem with remembering all of the tasks), speed, and no typos. Additional methods using text-mining analysis and machine learning to automatically complete some of the variables were also proposed, which would be another improvement. The analysis of the application of ASR in the conditions of an underground mine posed several challenges. A dictionary of specialized mining vocabulary was developed, and a source database was created, which improved the accuracy of speech-to-text transcription and the correctness of completed forms. Awareness of high noise disturbances in the underground mine made it necessary to conduct tests in places with high noise, and also in real conditions. The tests showed that noise has a slight impact on the correctness of transcription, which was considered a good starting point for further work and testing of noise-reduction methods. The final results show that the correctness of filling in the forms by people with varying degrees of experience with the application in mining conditions ranges from 70–80%, which is satisfactory. This, and the above conclusions, prove that the use of ASR to improve work records in the heavy machinery chamber kept by a foreman is possible.

## Figures and Tables

**Table 1:** Summary of tables and fields that are accessible by the foreman.

System	Table	No. of Variables	Data Types	No. of Data Type Occurrences
CMMS	Register of machines	14	Categorical	3
			[0–1]	7
			Numerical	4
	Register of entry and exit	11	Categorical	5
			Numerical	4
			Time	2
e-Raport	Machines working plan	3	Categorical	3
	Maintenance performed	13	Categorical	5
			[0–1]	3
			Numerical	2
			Text	3
	Settlement of work for machines	12	Categorical	3
			Numerical	8
			Text	1
	Settlement of work for employees	19	Categorical	13
			Numerical	2
			Time	3
			Text	1

**Table 2:** Description of fields that can be used by the voice assistant.

System	Table	Variables	Data Type	Explanation
CMMS	Register of machines	Machine	Categorical	Machine name
		PRD	[0–1]	Part of work spent in production
		DPL	[0–1]	Part of work spent on a planned service
		DNP	[0–1]	Part of work spent on an unplanned service
		NPP	[0–1]	Part of work spent on a planned repair
		NPL	[0–1]	Part of work spent on an unplanned repair
		NAW	[0–1]	Part of work spent on an emergency repair
		AWR	[0–1]	Part of work spent on an emergency stop
		Milometer value	Numerical	Milometer value
	Register of entry and exit	Company	Categorical	Company name
		Subcontracting company	Categorical	Name of the subcontracting company
		Name and surname	Categorical	Name and surname of the employee
eRaport	Machines working plan	Machine	Categorical	Machine name
		Operator	Categorical	Name and surname of the employee
		Mining department	Categorical	A mining department
	Maintenance performed	Machine	Categorical	Machine name
		Works to be done	Free written text	Description of the work to be performed on the current shift in free written text
		Operator	Categorical	Name and surname of the employee
		Mechanics	Categorical	Name and surname of the employee
		Work	Free written text	Listed elements for maintenance
		Work to be done on the next shift	Free written text	Description of the work to be performed on the next shift in free written text
		Milometer value	Numerical	Milometer value from machine
	Settlement of work for machines	Machine	Categorical	Machine names
		Operator	Categorical	Name and surname of the employee
		Mining department	Categorical	A mining department that requests a machine
	Settlement of work for employees	Employee	Categorical	Name, surname, HMC, employee ID
		Mining department	Categorical	A mining department that requests a machine
		Machine	Categorical	Machine name

**Table 3:** Review of the quality of algorithms on sample with different noises.

Noise Type	Noise	Well-Caught Words (%)	Word Error Rate	Levenshtein	Jaro	Jaro–Winkler
None	None	100	0	1.0	1.0	1.0
Normal Environment	Traffic jam	93.33	0.06	0.99	0.99	0.99
	Cleaning sounds	100.00	0.00	1.00	1.00	1.00
	Moving stuff	100.00	0.00	1.00	1.00	1.00
	Hair dryer	81.25	0.20	0.97	0.98	0.99
Industrial Noises	Electric motor	81.25	0.20	0.93	0.96	0.97
	Factory sounds	93.33	0.06	0.97	0.98	0.99
	Grinder	87.50	0.13	0.99	0.99	0.99
	Ship engine	73.33	0.26	0.92	0.96	0.98
	Car engine	87.50	0.13	0.98	0.99	0.99

**Table 4:** Categories prepared to train the model.

Category	Count
Drive system	2825
Fire extinguishing system	1900
Service brake system	1516
Electrical installation	699
Suspension arms and bushings	593
Hydraulic system	453
Central lubrication system	388
Running gear	329
Air conditioning system	319

**Table 5:** Confusion matrix for prepared model.

	Electrical Installation	Fire Extinguishing System	Suspension Arms and Bushings	Hydraulic System	Central Lubrication System	Running Gear	Air Conditioning System	Drive System	Service Brake System
Electrical installation	206	0	4	0	0	0	0	0	0
Fire extinguishing system	0	570	0	0	0	0	0	0	0
Suspension arms and bushings	6	0	172	0	0	0	0	0	0
Hydraulic system	0	0	1	115	0	0	0	0	0
Central lubrication system	0	0	0	0	136	0	0	0	0
Running gear	0	0	0	0	0	99	0	0	0
Air conditioning system	0	0	0	0	0	0	96	0	0
Drive system	3	0	14	0	0	1	1	828	0
Service brake system	0	0	0	0	0	0	0	0	455

**Table 6:** Table describing testers.

Person	Number of Recordings	Experience
P1	6	Device developer
P2	5	Had no previous contact with the device
P3	5	Knew the commands but had never used the device

**Table 7:** Results for each recording.

Person	Recording Place	Time	Avg. Entry Correctness (%)	Entries Fully Filled (%)
P1	1	02:25	89.58%	68.75%
P1	2	02:39	68.75%	56.25%
P1	3	02:53	87.50%	68.75%
P1	4	02:04	72.92%	50.00%
P1	5	02:21	87.50%	62.50%
P1	6	01:42	83.33%	62.50%
P2	7	01:28	75.00%	50.00%
P2	8	02:02	72.92%	50.00%
P2	9	01:47	81.25%	50.00%
P2	10	01:54	77.08%	50.00%
P2	11	01:42	85.42%	68.75%
P3	12	02:09	79.17%	50.00%
P3	13	02:00	83.33%	56.25%
P3	14	02:03	79.17%	50.00%
P3	15	02:21	79.17%	43.75%
P3	16	02:17	72.92%	43.75%

**Table 8:** Results for different data types.

The Type of the Variable	Accuracy
Machine	86.33%
Operator	92.58%
Department	60.16%

## Data Availability

Not applicable.
